# Supplementary material for: Novel Driver Strength Index highlights important cancer genes in TCGA PanCanAtlas patients
Source: PeerJ. 2022 Aug 11;10:e13860. doi: 10.7717/peerj.13860 (PMC9375969; doi:10.7717/peerj.13860)
Supplement: Supplemental Information 2 [file peerj-10-13860-s002.zip › COHORTS/genes plots/cohorts/2021_8_16_14_9_distribution_cohorts_females_SKCM.pdf]

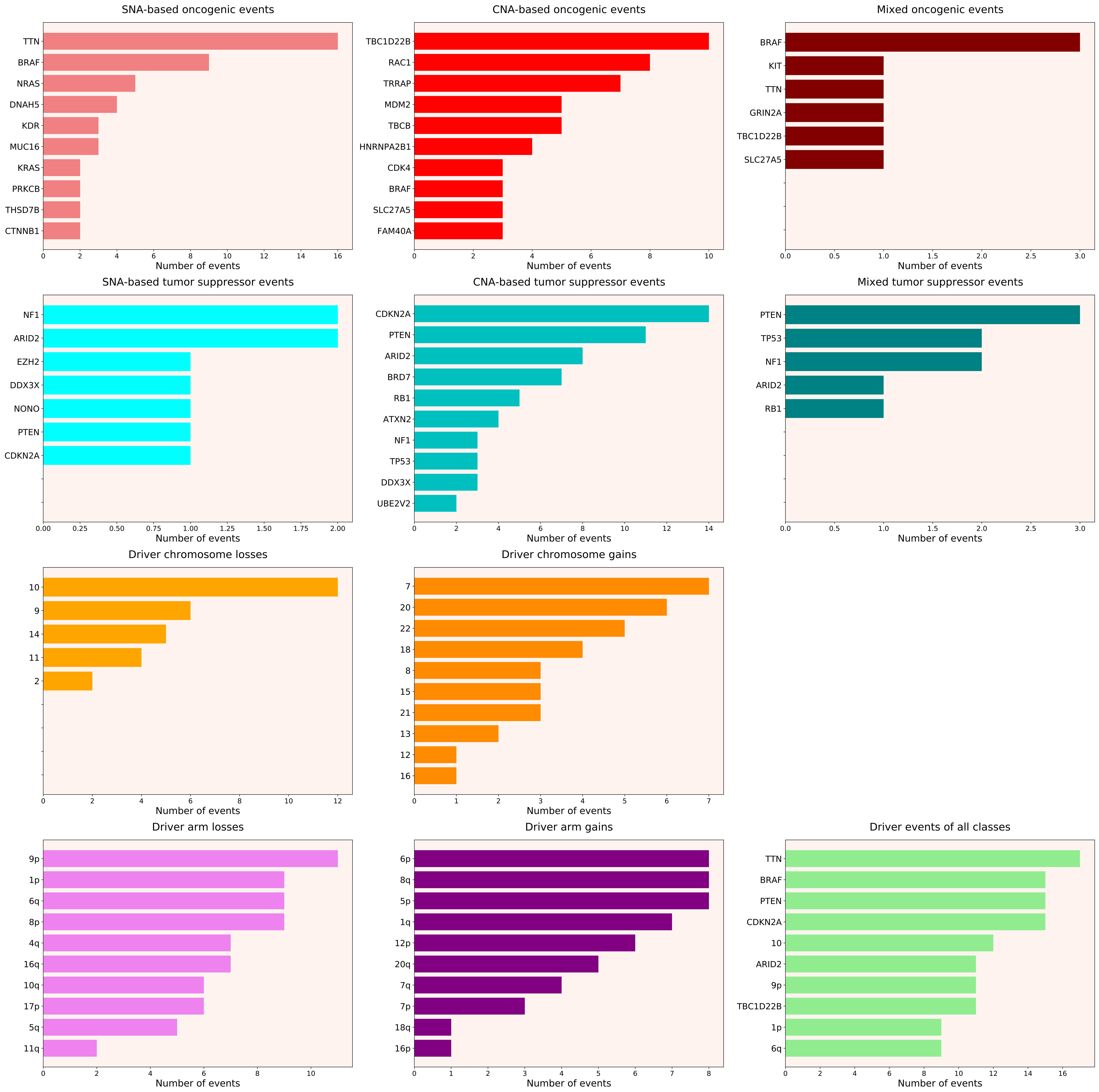

SNA-based oncogenic events
TTN
BRAF
NRAS
DNAH5
KDR
MUC16
KRAS
PRKCB
THSD7B
CTNNB1
Number of events
CNA-based oncogenic events
TBC1D22B
RAC1
TRRAP
MDM2
TBCB
HNRNPA2B1
CDK4
BRAF
SLC27A5
FAM40A
Number of events
Mixed oncogenic events
BRAF
KIT
TTN
GRIN2A
TBC1D22B
SLC27A5
Number of events
SNA-based tumor suppressor events
NF1
ARID2
EZH2
DDX3X
NONO
PTEN
CDKN2A
Number of events
CNA-based tumor suppressor events
CDKN2A
PTEN
ARID2
BRD7
RB1
ATXN2
NF1
TP53
DDX3X
UBE2V2
Number of events
Mixed tumor suppressor events
PTEN
TP53
NF1
ARID2
RB1
Number of events
Driver chromosome losses
10
9
14
11
2
Number of events
Driver chromosome gains
7
20
22
18
8
15
21
13
12
16
Number of events
Driver arm losses
9p
1p
6q
8p
4q
16q
10q
17p
5q
11q
Number of events
Driver arm gains
6p
8q
5p
1q
12p
20q
7q
7p
18q
16p
Number of events
Driver events of all classes
TTN
BRAF
PTEN
CDKN2A
10
ARID2
9p
TBC1D22B
1p
6q
Number of events
